# High glucose stimulates expression of aldosterone synthase (*CYP11B2*) and secretion of aldosterone in human adrenal cells

**DOI:** 10.1002/2211-5463.12277

**Published:** 2017-08-29

**Authors:** Hiroki Shimada, Naotaka Kogure, Erika Noro, Masataka Kudo, Kaori Sugawara, Ikuko Sato, Kyoko Shimizu, Makoto Kobayashi, Dai Suzuki, Rehana Parvin, Takako Saito‐Ito, Akira Uruno, Akiko Saito‐Hakoda, William E. Rainey, Sadayoshi Ito, Atsushi Yokoyama, Akira Sugawara

**Affiliations:** ^1^ Department of Molecular Endocrinology Tohoku University Graduate School of Medicine Sendai Miyagi Japan; ^2^ Division of Nephrology, Endocrinology and Vascular Medicine Tohoku University Graduate School of Medicine Sendai Miyagi Japan; ^3^ Department of Pediatrics Tohoku University Graduate School of Medicine Sendai Miyagi Japan; ^4^ Department of Medical Biochemistry Tohoku University Graduate School of Medicine Sendai Miyagi Japan; ^5^ Department of Molecular and Integrative Physiology University of Michigan Medical School Ann Arbor MI USA

**Keywords:** aldosterone synthase, diabetes mellitus, hypertension, NURR1

## Abstract

Aldosterone synthase is the key rate‐limiting enzyme in adrenal aldosterone production, and induction of its gene (*CYP11B2*) results in the progression of hypertension. As hypertension is a frequent complication among patients with diabetes, we set out to elucidate the link between diabetes mellitus and hypertension. We examined the effects of high glucose on *CYP11B2* expression and aldosterone production using human adrenal H295R cells and a stable H295R cell line expressing a *CYP11B2* 5′‐flanking region/luciferase cDNA chimeric construct. d‐glucose (d‐glu), but not its enantiomer l‐glucose, dose dependently induced *CYP11B2* transcription and mRNA expression. A high concentration (450 mg·dL^−1^) of d‐glu time dependently induced *CYP11B2* transcription and mRNA expression. Moreover, high glucose stimulated secretion of aldosterone into the media. Transient transfection studies using deletion mutants/nerve growth factor‐induced clone B (NGFIB) response element 1 (NBRE‐1) point mutant of *CYP11B2* 5′‐flanking region revealed that the NBRE‐1 element, known to be activated by transcription factors NGFIB and NURR1, was responsible for the high glucose‐mediated effect. High glucose also induced the mRNA expression of these transcription factors, especially that of NURR1, but NURR1 knockdown using its siRNA did not affect high glucose‐induced *CYP11B2* mRNA expression. Taken together, it is speculated that high glucose may induce *CYP11B2* transcription via the NBRE‐1 element in its 5′‐flanking region, resulting in the increase in aldosterone production although high glucose‐induced NURR1 is not directly involved in the effect. Additionally, glucose metabolism and calcium channels were found to be involved in the high glucose effect. Our observations suggest one possible explanation for the high incidence of hypertension in patients with diabetes.

**Abbreviations**
ARB: angiotensin II receptor blocker
ATF: activating transforming factor
CCB: calcium channel blocker
COUP‐TF: chicken ovalbumin upstream promoter transcription factor
CREB: cAMP‐response element binding protein
CREM: cAMP‐response element modulator
NBRE: NGFIB response element
NGFIB: nerve growth factor‐induced clone B
NURR1: Nur‐related factor 1
SF‐1: steroidogenic factor‐1

The number of patients with diabetes mellitus is increasing every year, and 382 million people in the world were estimated to be affected in 2013 1. Among patients with diabetes, hypertension is one of the most frequently observed complications. In Japan, the incidence of hypertension in patients with diabetes is approximately 60%, which is twice that in nondiabetic people 2. The etiology of hypertension in patients with diabetes is partially explained by the effect of hyperinsulinemia on renal proximal tubules due to insulin resistance 3. Additionally, endothelial dysfunction and atherosclerosis induced by diabetes mellitus may also contribute to the progression of hypertension 4. However, the direct involvement of high glucose on the etiology of hypertension in patients with diabetes still remains uncertain.

The renin–angiotensin–aldosterone system (RAAS) is known as the main humoral pathway involved in the etiology of hypertension, and aldosterone, the final product of the pathway, plays an important role in the progression of hypertension and vascular damages in combination with sodium 5. Aldosterone is synthesized in the zona glomerulosa of the adrenal cortex from cholesterol catalyzed via side chain cleavage enzyme (CYP11A1), 3β‐hydroxysteroid dehydrogenase (3β‐HSD), steroid 21‐hydroxylase (CYP21), and aldosterone synthase (CYP11B2), which is the key rate‐limiting enzyme in aldosterone production 6. Aldosterone synthase gene (*CYP11B2*) expression is mainly regulated by angiotensin II (AII) and potassium via transcription factors including Nur‐related factor 1 (NURR1) 7. Recently, genetic analyses of *KCNJ5*,* ATP1A1*,* ATP2B3*, and *CACNA1D* have revealed that chronic overexpression of *CYP11B2* induces not only aldosterone hypersecretion but also the formation of primary aldosteronism 8, resulting in the progression of severe hypertension. Moreover, aberrant WNT signaling caused by mutations in *CTNNB1* has also been recognized to be involved in the formation of primary aldosteronism 9. In order to investigate the direct link between hypertension and diabetes mellitus, we here examined the effects of high glucose on *CYP11B2* expression and aldosterone secretion using human adrenal H295R cells.

## Materials and methods

### Reagents

d‐Glucose (d‐glu) was purchased from Wako (Osaka, Japan), and l‐glucose (l‐glu), used for the adjustment of osmolality, was purchased from Sigma (St. Louis, MO, USA). 2‐Deoxy‐d‐glu, d‐sorbitol, d‐fructose, and 3‐*O*‐methyl‐d‐glu were purchased from Sigma. Olmesartan (olmesartan medoxomil) was purchased from Toronto Research Chemicals (North York, Canada). Losartan (losartan potassium) was purchased from LKT Laboratories (St. Paul, MN, USA). Valsartan was purchased from Cayman Chemical (Ann Arbor, MI, USA). Candesartan (trityl candesartan cilexetil) was purchased from Sequoia Research Products (Pangbourne, UK). Nifedipine and efonidipine (efonidipine hydrochloride monoethanolate) were purchased from Sigma. Amlodipine was purchased from Cayman Chemical. Benidipine (benidipine hydrochloride) was kindly provided by Kyowa Hakko Kirin Pharma (Tokyo, Japan). Human AII was purchased from Sigma.

### Plasmids

Subcloned chimeric constructs containing the human *CYP11B2* genomic DNA and luciferase cDNA (pGL3‐Basic; Promega, Madison, WI, USA) 7, 10 were used for the transient transfection studies: −1521/+2‐luc (harboring the *CYP11B2* 5′‐flanking region from −1521 to +2 relative to the transcription start site upstream of the luciferase cDNA in pGL3‐Basic), −747/+2‐luc; −135/+2‐luc; −106/+2‐luc; −65/+2‐luc. The nerve growth factor‐induced clone B (NGFIB) response element 1 (NBRE‐1) mutant construct of −1521/+2‐luc (NBRE‐1 mut) was also used 11. In some experiments, a previously described stable H295R cell line expressing *CYP11B2* promoter (−1521/+2)/luciferase chimeric reporter construct (*CYP11B2*‐H295R cells) was used 7. β‐Galactosidase control plasmid in pCMV (pCMV‐β‐gal) was purchased from Clontech (Palo Alto, CA, USA).

### Cell culture

H295R cells or *CYP11B2*‐H295R cells were grown with a 1 : 1 mixture of Dulbecco's modified Eagle's medium and Ham's F12 medium supplemented with 10% fetal bovine serum (FBS), Insulin‐Transferrin‐Selenium‐G Supplements (Invitrogen, Carlsbad, CA, USA), 1.25 mg·mL^−1^ BSA (Sigma), 5.35 μg·mL^−1^ linoleic acid (Sigma), 100 U·mL^−1^ penicillin, and 100 μg·mL^−1^ streptomycin. Cells were cultured in a humidified incubator at 37 °C with 5% CO_2_. As the d‐glu concentration in the media was ~ 100 mg·dL^−1^, we added either concentrated d‐glu or l‐glu solution to adjust the final concentration. For example, the 450 mg·dL^−1^ d‐glu concentration was composed of 100 mg·dL^−1^ d‐glu from the media and 350 mg·dL^−1^ d‐glu from the concentrated d‐glu solution, and its osmolality‐adjusted control was composed of 100 mg·dL^−1^ d‐glu from the media and 350 mg·dL^−1^ l‐glu from the concentrated l‐glu solution. In some experiments, 2‐deoxy‐d‐glu, 3‐*O*‐methyl‐d‐glu, d‐sorbitol, or d‐fructose was used instead of l‐glu. Moreover, *CYP11B2*‐H295R cells were incubated either with angiotensin II receptor blockers (ARBs) or with calcium channel blockers (CCBs) in the presence of 450 mg·dL^−1^ d‐glu.

### RNA Preparation and quantitative real‐time PCR

When H295R cells were grown to 60% confluence in 24‐multiwell plates, they were exposed to several concentrations of d‐glu or d‐glu plus l‐glu for the indicated times, and their total RNA was extracted using Sepasol®‐RNA I Super G (Nacalai Tesque, Kyoto, Japan) according to the manufacturer's instructions. Total RNA were subjected to reverse transcription (RT) reaction using PrimeScript Reverse Transcriptase (Takara Bio, Ohtsu, Japan) with random 6‐mer and oligo dT primers according to the manufacturer's instructions. Thereafter, the obtained templates were used for quantitative real‐time PCR (95 °C, 3 min for one cycle; 95 °C, 15 s; 60 °C, 10 s; 72 °C, 20 s for 40 cycles) either with iQ Supermix (Bio‐Rad, Hercules, CA, USA; for *CYP11B2*,* CYP11B1*,* HSD3B2*, and *CYP17*) or with THUNDERBIRD® SYBR® qPCR Mix (TOYOBO, Osaka, Japan; for others) by DNA Engine thermal cycler attached to Chromo4 detector (Bio‐Rad). The sequences of the primers and TaqMan probes are shown in Table 1.

**Table 1:** The sequences of primers (A) and TaqMan probes (B) used for quantitative real‐time PCR

A		
	Sense primer (5′–3′)	Antisense primer (3′–5′)
CYP11B2	GGCAGAGGCAGAGATGCTG	CTTGAGTTAGTGTCTCCACCAGGA
CYP11B1	GGCAGAGGCAGAGATGCTG	TCTTGGGTTAGTGTCTCCACCTG
STAR	GGAGATCAAGGTCCTGCAGA	CACGCTCACAAAGTCACGG
CYP11A1	TTCCGCTTTGCCTTTGAGTC	TGGCATCAATGAATCGCTGG
HSD3B2	GCGGCTAATGGGTGGAATCTA	CATTGTTGTTCAGGGCCTCAT
CYP21A2	AGACTACTCCCTGCTCTGGA	CTCATGCGCTCACAGAACTC
CYP17	CAGAATGTGGGTTTCAGCCG	CTCACCGATGCTGGAGTCAA
NURR1	AGAGAAGATCCCTGGCTTCG	CAAGACCACCCCATTGCAAAA
NGFIB	CCTGGAGCTCTTCATCCTCC	TGTCAATCCAGTCCCCGAAG
CREB	GGAGCTTGTACCACCGGTAA	GGGCTAATGTGGCAATCTGT
CREM	C AG AAG AAG C AACAC G C AAA	CAGCCACACGATTTTCAAGA
ATF‐1	GAAGATACACGGGGCAGAAA	CTTGCCAACTGTAAGGCTCC
ATF‐2	ACCATGCCTGTTGCTATTCC	CCTGGAACACTAGGCACCAT
SF‐1	GAGAGCCAGAGCTGCAAGAT	CTTGTACATCGGCCCAAACT
COUP‐TF	TCAAAGCCATCGTGCTGTTC	GTACTGGCTCCTCACGTACT
β‐actin	CCAACCGCGAGAAGATGACC	CCAGAGGCGTACAGGGATAG
GAPDH	ATCCCATCACCATCTTCCAG	ATGAGTCCTTCCACGATACC

B	
	Taqman probe
CYP11B2	[6‐FAM]CTGCACCACGTGCTGAAGCACT[TAMRA‐6‐FAM]
CYP11B1	[6‐FAM]TGCTGCACCATGTGCTGAAACACCT[TAMRA‐6‐FAM]
HSD3B2	[6‐FAM]TGATACCTTGTACACTTGTGC[TAMRA‐6‐FAM]
CYP17	[6‐FAM]TCAGTGACCGTAACCGTCTC[TAMRA‐6‐FAM]

### Transient transfection and luciferase assay

H295R cells were plated to 60% confluence in 24‐multiwell plates. Thereafter, they were transiently transfected with 200 ng luciferase reporter plasmids and 100 ng pCMV‐β‐gal using Lipofectamine® 2000 Transfection Reagent (Life Technologies, Carlsbad, CA, USA) for 24 h. The cells were then exposed to d‐glu or l‐glu for the indicated times and concentrations. They were thereafter washed with PBS, and the cell extracts were prepared using Glo Lysis Buffer (Promega). Luciferase activity was measured using Bright‐Glo reagents (Promega), and β‐galactosidase activity was simultaneously measured. Data were normalized by the β‐galactosidase activities. When the stable *CYP11B2*‐H295R cells 7 were used, only the luciferase activity was measured.

### Small interfering RNA transfection

Small interfering RNA (siRNA) for NURR1 (s9785) 12 was obtained from Thermo Fisher Scientific (Waltham, MA, USA), and negative control siRNA (SI03650318) was obtained from Qiagen (Hilden, Germany). H295R cells were plated to 60% confluence in 12‐multiwell plates. Thereafter, they were transiently transfected with 10 pmol of each siRNA by electroporation using Nucleofector 4D™ (Lonza, Basel, Switzerland) as previously described 13.

### Measurement of aldosterone/cortisol concentration

H295R cells were plated to 60% confluence in 24‐multiwell plates. Thereafter, they were exposed to either 100 mg·dL^−1^ d‐glu, 450 mg·dL^−1^ d‐glu, 100 mg·dL^−1^ d‐glu plus 100 nmol·L^−1^ AII (for aldosterone), or 450 mg·dL^−1^ d‐glu plus 100 nmol·L^−1^ AII (for aldosterone) for 72 h. The aldosterone and cortisol concentrations of the media were thereafter measured by Aldosterone EIA Kit and Cortisol EIA Kit (Cayman Chemical), respectively, after their extraction with dichloromethane according to the manufacturer's instructions. The obtained data were normalized by the protein concentrations measured by Protein Assay Kit (Bio‐Rad).

### Statistical analyses

All data are presented as mean ± SEM. For the statistical analyses, ANOVA followed by post hoc Tukey's test was performed. *P* < 0.05 was considered statistically significant.

## Results

### Effects of high glucose on CYP11B2 expression and aldosterone secretion

**Figure 1:**
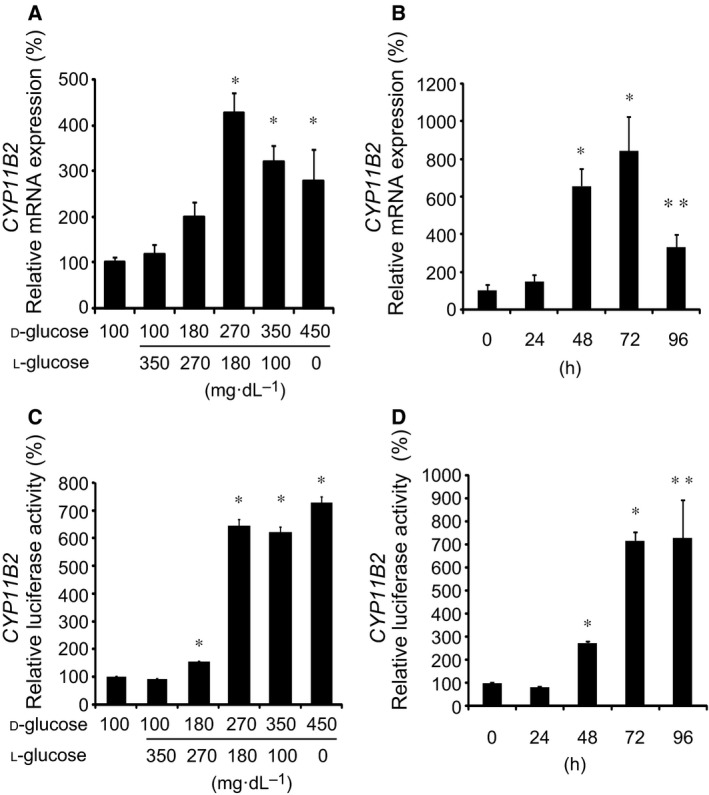
Effects of high glucose on *CYP11B2* transcription and mRNA expression. (A) Dose–response effects of high glucose on *CYP11B2* mRNA expression. H295R cells were incubated with either 100 mg·dL^−1^ d‐glu, 100 mg·dL^−1^ d‐glu plus 350 mg·dL^−1^ l‐glu, 180 mg·dL^−1^ d‐glu plus 270 mg·dL^−1^ l‐glu, 270 mg·dL^−1^ d‐glu plus 180 mg·dL^−1^ l‐glu, 350 mg·dL^−1^ d‐glu plus 100 mg·dL^−1^ l‐glu, or 450 mg·dL^−1^ d‐glu for 48 h. Data represent mean ± SEM (*n* = 4), percentage of 100 mg·dL^−1^ d‐glu (control), normalized by β‐actin mRNA levels. (B) Time‐course effects of high glucose on *CYP11B2* mRNA expression. H295R cells were incubated with 450 mg·dL^−1^ d‐glu for the indicated times. Data represent mean ± SEM (*n* = 4), percentage of 0 h (control), normalized by β‐actin mRNA levels. (C) Dose–response effects of high glucose on *CYP11B2* transcription. *CYP11B2*‐H295R cells were incubated with several concentrations of d‐glu as in (A) for 48 h. Data represent mean ± SEM (*n* = 4), percentage of 100 mg·dL^−1^ d‐glu (control). (D) Time‐course effects of high glucose on *CYP11B2* transcription. *CYP11B2*‐H295R cells were incubated with 450 mg·dL^−1^ d‐glu for the indicated times. Data represent mean ± SEM (*n* = 4), percentage of 0 h (control). (A‐D) * *P* < 0.01, vs. control. ** *P* < 0.05, vs. control.

**Figure 2:**
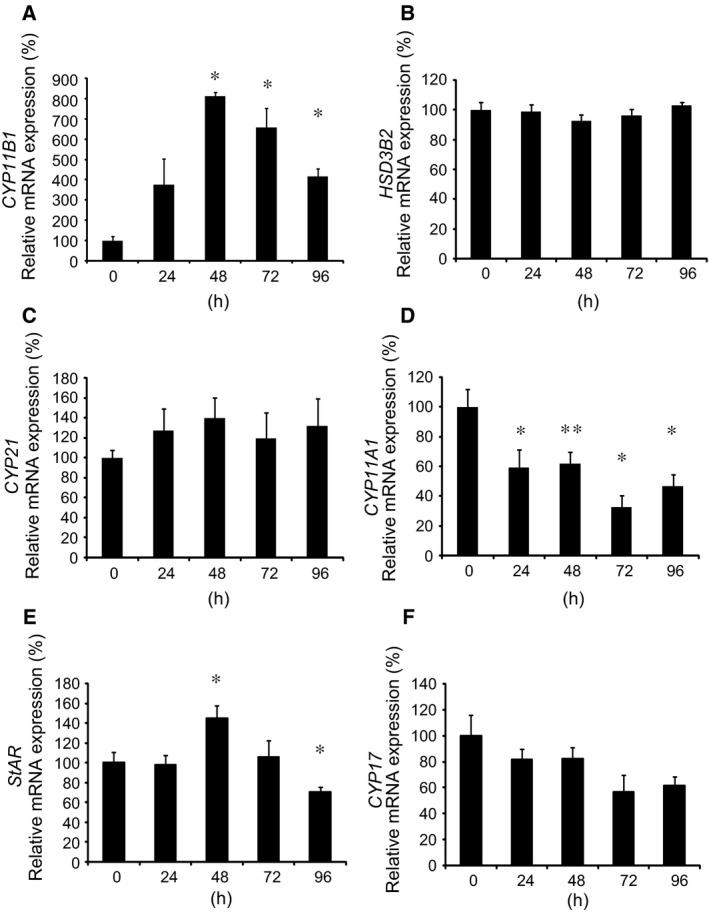
Effects of high glucose on mRNA expression of enzymes/protein involved in adrenal steroidogenesis. Effects of high glucose on *CYP11B1* mRNA expression (A), *HSD3B2* mRNA expression (B), *CYP21* mRNA expression (C), *CYP11A1* mRNA expression (D), *StAR* mRNA expression (E), and *CYP17* mRNA expression (F). H295R cells were incubated with 450 mg·dL^−1^ d‐glu for the indicated times. Data represent mean ± SEM (*n* = 4), percentage of 0 h (control), normalized by β‐actin mRNA levels. (A,D,E) *P < 0.01, vs. control. **P < 0.05, vs. control.

**Figure 3:**
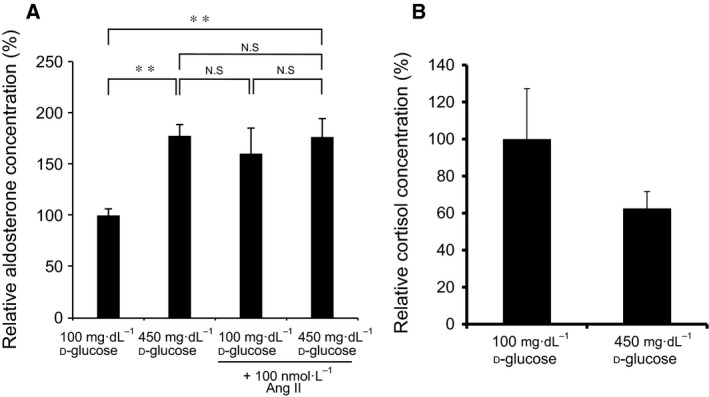
Effects of high glucose on aldosterone and cortisol secretion. (A) Effects of high glucose and/or AII on aldosterone secretion. H295R cells were incubated with either 100 mg·dL^−1^ d‐glu, 450 mg·dL^−1^ d‐glu, 100 mg·dL^−1^ d‐glu plus 100 nmol·L^−1^ AII, or 450 mg·dL^−1^ d‐glu plus 100 nmol·L^−1^ AII for 72 h. Data represent mean ± SEM (*n* = 4), percentage of 100 mg·dL^−1^ d‐glu (control), normalized by the protein concentrations. (B) Effects of high glucose on cortisol secretion. H295R cells were incubated with either 100 mg·dL^−1^ d‐glu or 450 mg·dL^−1^ d‐glu for 72 h. Data represent mean ± SEM (*n* = 4), percentage of 100 mg·dL^−1^ d‐glu (control), normalized by the protein concentrations. Ang II; AII. (A) ** *P* < 0.05, vs. control.

We first examined the effects of high glucose on *CYP11B2* mRNA expression using H295R cells. As shown in Fig. 1A, d‐glu levels above 270 mg·dL^−1^ significantly induced *CYP11B2* mRNA expression. Time‐course experiments in the presence of 450 mg·dL^−1^ d‐glu demonstrated that high glucose induced *CYP11B2* mRNA expression after 48 h (Fig. 1B). We next examined the effect of high glucose on *CYP11B2* transcription using stable *CYP11B2*‐H295R cells 7, and also observed similar stimulatory effects in both the dose–response (Fig. 1C) and time‐course (Fig. 1D) experiments. We also examined the effect of high glucose (450 mg·dL^−1^ d‐glu) on the mRNA expression of other enzymes/protein involved in adrenal steroidogenesis. As shown in Fig. 2, high glucose treatment significantly induced the mRNA expression of 11β‐hydroxylase gene (*CYP11B1*) (A) and steroidogenic acute regulatory protein gene (*StAR*) (E) after 48‐h incubation, while high glucose treatment significantly decreased that of *CYP11A1* (D) after 24‐h incubation. High glucose treatment did not affect the mRNA expression of 3β‐HSD gene (*HSD3B2*) (B) and *CYP21* (C), while it tended to decrease, although not significantly, the expression of 17α‐hydroxylase/17,20 lyase gene (*CYP17*) (F). We then examined the effect of high glucose on aldosterone secretion from H295R cells. As shown in Fig. 3A, incubation of the cells in the presence of 450 mg·dL^−1^ d‐glu for 72 h significantly induced aldosterone secretion into the media, which was comparable to the AII‐induced aldosterone secretion. Incubation with 450 mg·dL^−1^ d‐glu plus AII did not further increase the aldosterone secretion (Fig. 3A). It can be concluded that high glucose induces *CYP11B2* transcription and mRNA expression resulting in the increase in aldosterone secretion. In contrast, although high glucose induced the mRNA expression of *CYP11B1* (Fig. 2A), it did not induce cortisol secretion into the media (Fig. 3B) probably due to the decreasing trend of *CYP17* mRNA expression (Fig. 2F).

### Identification of the element(s) responsible for the high glucose‐induced CYP11B2 transactivation

**Figure 4:**
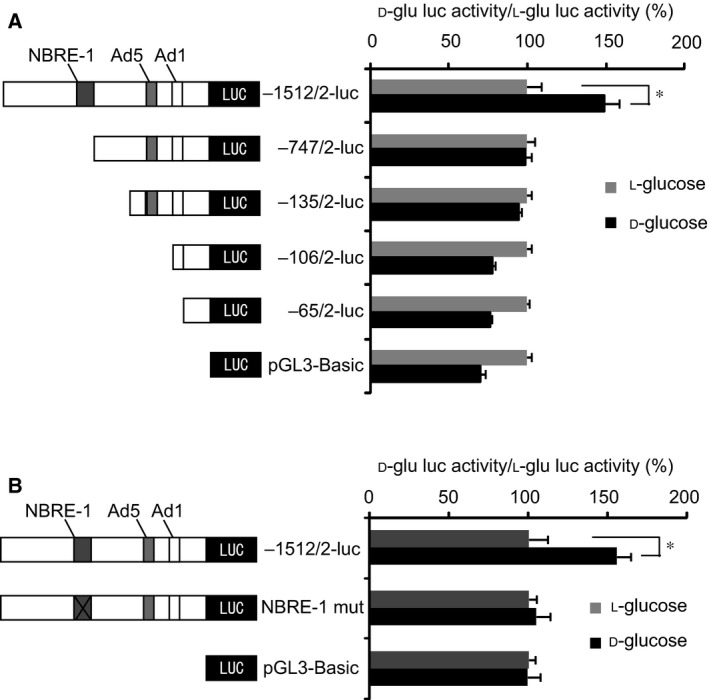
Effects of 5′‐flanking region mutants on the high glucose‐induced *CYP11B2* transactivation. (A) Effects of *CYP11B2* 5′‐flanking region deletion mutants. Either −1521/+2‐luc, −747/+2‐luc, −135/+2‐luc, −106/+2‐luc, −65/+2‐luc, or pGL3‐Basic (control plasmid) was transiently transfected with pCMV‐β‐gal into H295R cells, and the cells were incubated either with 450 mg·dL^−1^ d‐glu or with 100 mg·dL^−1^ d‐glu plus 350 mg·dL^−1^ l‐glu for 48 h. Data represent mean ± SEM (*n* = 4), percentage of control (l‐glu), normalized by β‐galactosidase activities. (B) Effects of NBRE‐1 point mutant. Either −1521/+2‐luc, NBRE‐1 mut, or pGL3‐Basic (control plasmid) was transiently transfected with pCMV‐β‐gal into H295R cells, and the cells were incubated either with 450 mg·dL^−1^ d‐glu or with 100 mg·dL^−1^ d‐glu plus 350 mg·dL^−1^ l‐glu for 48 h. Data represent mean ± SEM (*n* = 4), percentage of control (l‐glu), normalized by β‐galactosidase activities. (A, B) *P < 0.01, vs. control.

In order to identify the element(s) responsible for the high glucose‐induced *CYP11B2* transactivation, we examined the effects of high glucose on the promoter activity of *CYP11B2* 5′‐flanking region deletion mutants by comparing the effects between d‐glu (450 mg·dL^−1^) and l‐glu (100 mg·dL^−1^ d‐glu and 350 mg·dL^−1^ l‐glu) using H295R cells. As shown in Fig. 4A, although high glucose‐induced *CYP11B2* transactivation was significantly observed in −1521/+2‐luc, it was not observed in −747/+2‐luc, −135/+2‐luc, −106/+2‐luc, or −65/+2‐luc. These data indicate that the region between −1521 and −747 may be responsible for the high glucose effect. As NBRE‐1 element 11, which is known to be transactivated by NURR1 and NGFIB 11, 14, is located within the region (−766/−759), we next examined the effect of high glucose on the element. As shown in Fig. 4B, point mutation of NBRE‐1 element (NBRE‐1 mut) completely suppressed the high glucose effect. These data indicate that the element responsible for the high glucose‐induced *CYP11B2* transactivation may possibly be the NBRE‐1 element.

### Effects of high glucose on the expression of transcription factors involved in CYP11B2 transcription

**Figure 5:**
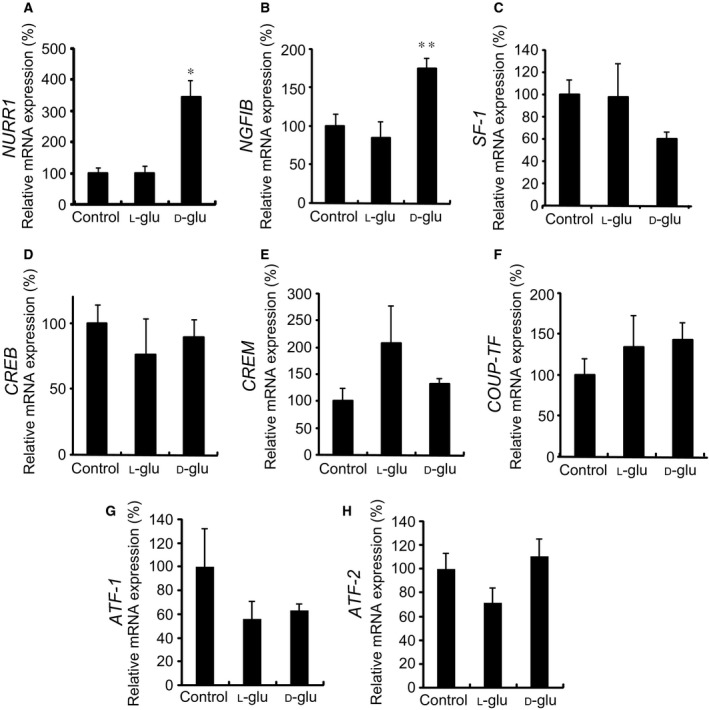
Effects of high glucose on mRNA expression of transcription factors. Effects of high glucose on NURR1 mRNA expression (A), NGFIB mRNA expression (B), SF‐1 mRNA expression (C), CREB mRNA expression (D), CREM mRNA expression (E), COUP‐TF mRNA expression (F), ATF‐1 mRNA expression (G), and ATF‐2 mRNA expression (H). H295R cells were incubated with either 100 mg·dL^−1^ d‐glu (control), 100 mg·dL^−1^ d‐glu plus 350 mg·dL^−1^ l‐glu, or 450 mg·dL^−1^ d‐glu for 48 h. Data represent mean ± SEM (*n* = 4), percentage of 100 mg·dL^−1^ d‐glu (control), normalized by the β‐actin mRNA levels. (A) *P < 0.01, vs. control. (B) **P < 0.05, vs. control.

We next examined the effects of high glucose on the mRNA expression of transcription factors that are known to regulate *CYP11B2* promoter 14 using H295R cells. As shown in Fig. 5A, d‐glu (450 mg·dL^−1^), but not control (100 mg·dL^−1^ d‐glu) or l‐glu (100 mg·dL^−1^ d‐glu and 350 mg·dL^−1^ l‐glu), significantly induced the expression of NURR1 mRNA. d‐glu also induced the mRNA expression of NGFIB (Fig. 5B), but not that of steroidogenic factor‐1 (SF‐1; Fig. 5C), cAMP‐response element binding protein (CREB; Fig. 5D), cAMP‐response element modulator (CREM; Fig. 5E), chicken ovalbumin upstream promoter transcription factor (COUP‐TF; Fig. 5F), activating transforming factor‐1 (ATF‐1; Fig. 5G), or ATF‐2 (Fig. 5H). As NURR1 is known to bind to NBRE‐1 and activate it 11, it is indicated that high glucose‐induced NURR1 may transactivate *CYP11B2* expression via the NBRE‐1 element.

### Effects of NURR1 siRNA on the high glucose‐induced CYP11B2 mRNA expression

**Figure 6:**
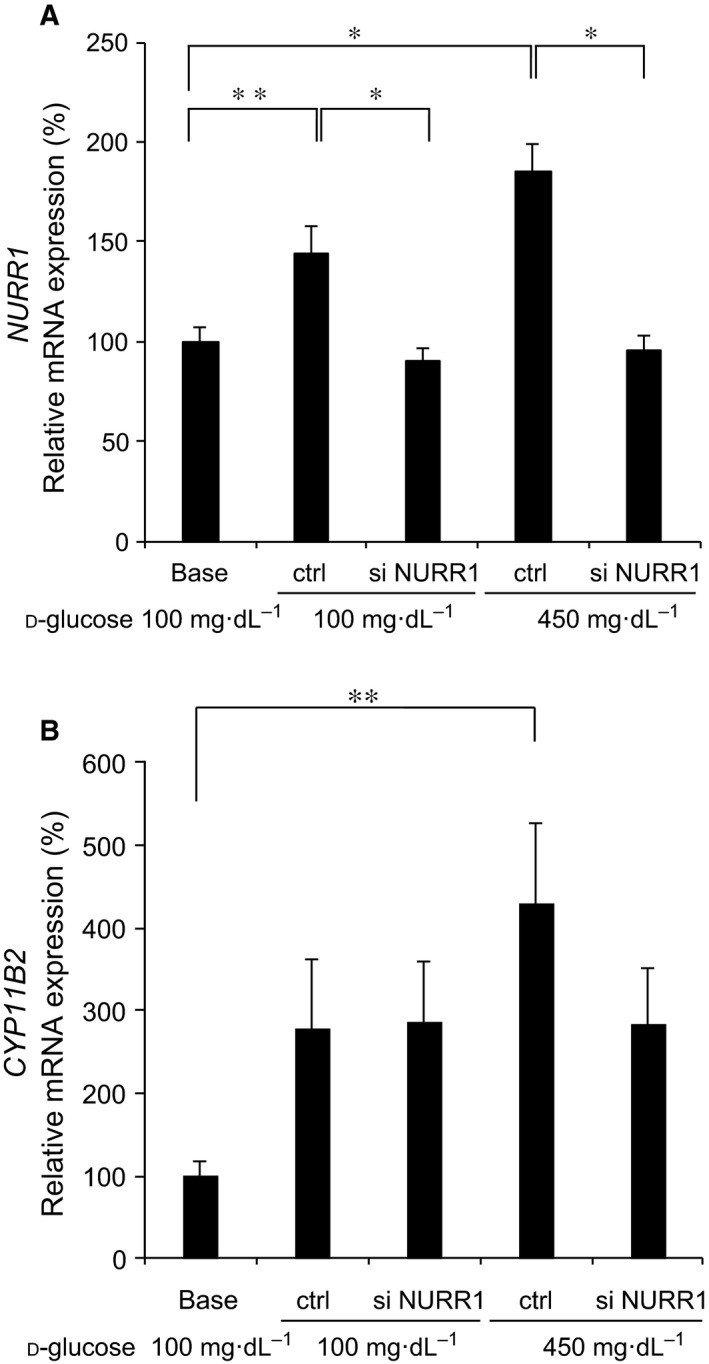
Effects of NURR1 siRNA. Effects of NURR1 siRNA on NURR1 mRNA expression (A) and *CYP11B2* mRNA expression (B). H295R cells transfected either with control siRNA (ctrl) or with NURR1 siRNA (si NURR1) were incubated with either 100 mg·dL^−1^ d‐glu or 450 mg·dL^−1^ d‐glu for 48 h. In some experiments, untransfected H295R cells were incubated with 100 mg·dL^−1^ d‐glu for 48 h (base). Data represent mean ± SEM (*n* = 11), percentage of 100 mg·dL^−1^ d‐glu (base), normalized by GAPDH mRNA levels. (A,B) *P < 0.01. **P < 0.05.

In order to examine the involvement of NURR1 in the high glucose‐induced *CYP11B2* mRNA expression, we next transfected either the control or NURR1 siRNA into H295R cells, and thereafter treated the cells with 450 mg·dL^−1^ d‐glu for 48 h. As shown in Fig. 6A, NURR1 mRNA expression was significantly decreased by the transfection of NURR1 siRNA as compared to that of control siRNA in the presence of either 100 mg·dL^−1^ d‐glu or 450 mg·dL^−1^ d‐glu, suggesting the efficient knockdown of endogenous NURR1 mRNA. However, NURR1 knockdown by its siRNA transfection did not affect the high glucose‐induced *CYP11B2* mRNA expression in comparison with control siRNA transfection (Fig. 6B). These data indicate that other NR4A family members or other transcription factors may be involved in the high glucose‐induced *CYP11B2* mRNA expression via the NBRE‐1 element.

### Effects of 2‐deoxy‐d‐glucose, 3‐*O*‐methyl‐d‐glucose, d‐sorbitol, and d‐fructose on CYP11B2 mRNA expression

**Figure 7:**
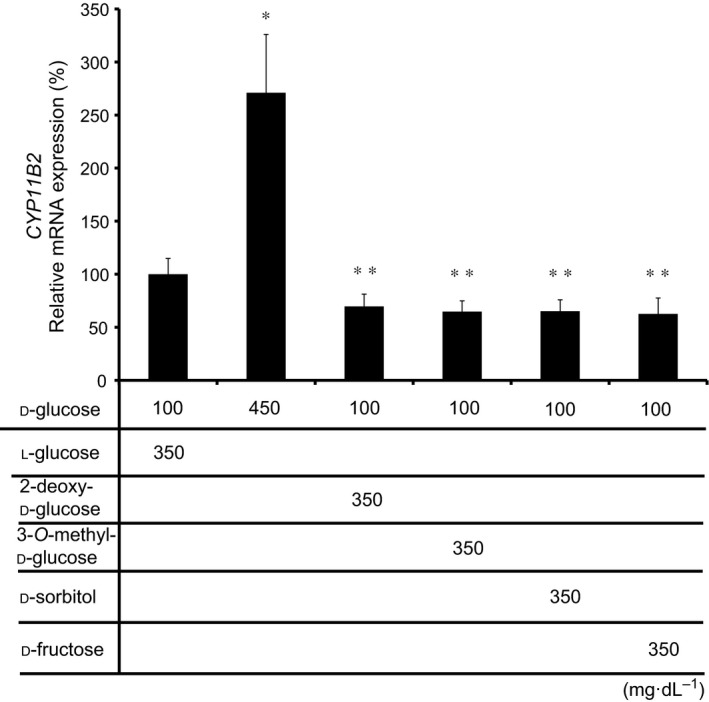
Effects of 2‐deoxy‐d‐glu, 3‐*O*‐methyl‐d‐glu, d‐sorbitol, and d‐fructose on *CYP11B2* mRNA expression. H295R cells were incubated with either 100 mg·dL^−1^ d‐glu plus 350 mg·dL^−1^ l‐glu, 450 mg·dL^−1^ d‐glu, 100 mg·dL^−1^ d‐glu plus 350 mg·dL^−1^ 2‐deoxy‐d‐glu, 100 mg·dL^−1^ d‐glu plus 350 mg·dL^−1^ 3‐*O*‐methyl‐d‐glu, 100 mg·dL^−1^ d‐glu plus 350 mg·dL^−1^ d‐sorbitol, or 100 mg·dL^−1^ d‐glu plus 350 mg·dL^−1^ d‐fructose for 72 h. Data represent mean ± SEM (*n* = 4), percentage of 100 mg·dL^−1^ d‐glu plus 350 mg·dL^−1^ l‐glu, normalized by the β‐actin mRNA levels. *P < 0.01, vs. 100 mg·dL^−1^ d‐glu plus 350 mg·dL^−1^ l‐glu. **P < 0.01, vs. 450 mg·dL^−1^ d‐glu.

We next examined the involvement of glucose metabolism on the high glucose‐induced *CYP11B2* expression. When we treated H295R cells with either 2‐deoxy‐d‐glu, which could be phosphorylated but could not be metabolized further 15, 16, or 3‐*O*‐methyl‐d‐glu, which could not be phosphorylated 16, the induction of *CYP11B2* mRNA expression was not observed (Fig. 7). These data suggest that d‐glu metabolization may be more necessary for the induction than glucose 6‐phosphate. Moreover, incubation with d‐sorbitol or d‐fructose, both of which are d‐glu metabolites via the polyol pathway 17, did not affect *CYP11B2* mRNA expression (Fig. 7), indicating that the pathway may not be involved in the induction.

### Effects of ARBs and CCBs on the high glucose‐induced CYP11B2 transcription

**Figure 8:**
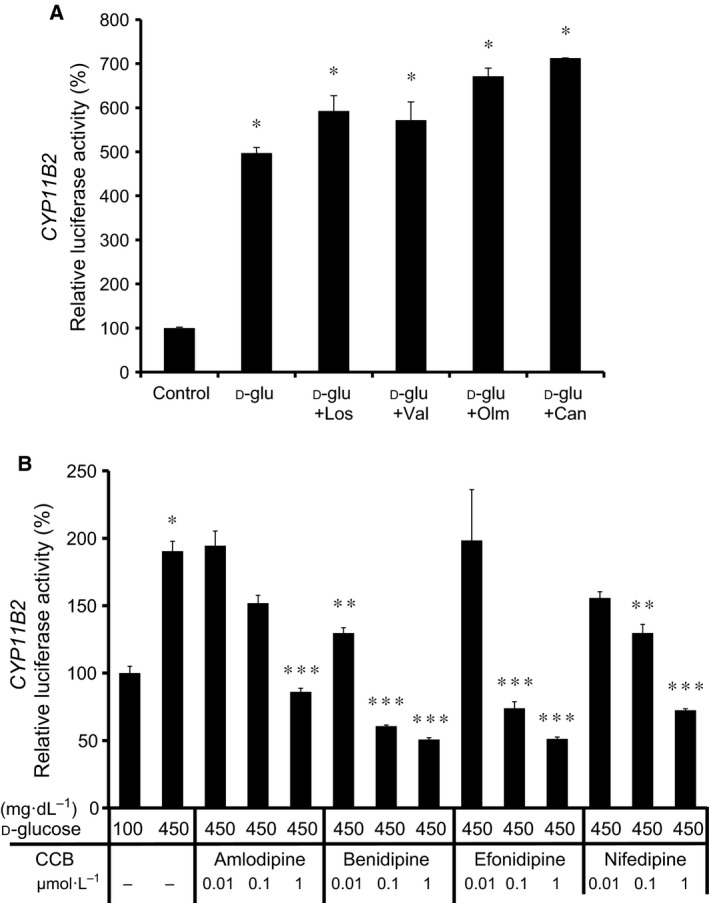
Effects of ARBs (A) and CCBs (B) on the high glucose‐induced *CYP11B2* transactivation. In (A), *CYP11B2*‐H295R cells were incubated with either 100 mg·dL^−1^ d‐glu (control), 450 mg·dL^−1^ d‐glu (d‐glu), 450 mg·dL^−1^ d‐glu plus 1 μmol·L^−1^ losartan (d‐glu + Los), 450 mg·dL^−1^ d‐glu plus 1 μmol·L^−1^ valsartan (d‐glu + Val), 450 mg·dL^−1^ d‐glu plus 1 μmol·L^−1^ olmesartan (d‐glu + Olm), or 450 mg·dL^−1^ d‐glu plus 1 μmol·L^−1^ candesartan (d‐glu + Can) for 48 h. Data represent mean ± SEM (*n* = 3), percentage of control. *P < 0.01, vs. control. In (B), *CYP11B2*‐H295R cells were incubated with either 100 mg·dL^−1^ d‐glu, 450 mg·dL^−1^ d‐glu, 450 mg·dL^−1^ d‐glu plus 0.01 μmol·L^−1^ amlodipine, 450 mg·dL^−1^ d‐glu plus 0.1 μmol·L^−1^ amlodipine, 450 mg·dL^−1^ d‐glu plus 1 μmol·L^−1^ amlodipine, 450 mg·dL^−1^ d‐glu plus 0.01 μmol·L^−1^ benidipine, 450 mg·dL^−1^ d‐glu plus 0.1 μmol·L^−1^ benidipine, 450 mg·dL^−1^ d‐glu plus 1 μmol·L^−1^ benidipine, 450 mg·dL^−1^ d‐glu plus 0.01 μmol·L^−1^ efonidipine, 450 mg·dL^−1^ d‐glu plus 0.1 μmol·L^−1^ efonidipine, 450 mg·dL^−1^ d‐glu plus 1 μmol·L^−1^ efonidipine, 450 mg·dL^−1^ d‐glu plus 0.01 μmol·L^−1^ nifedipine, 450 mg·dL^−1^ d‐glu plus 0.1 μmol·L^−1^ nifedipine, or 450 mg·dL^−1^ d‐glu plus 1 μmol·L^−1^ nifedipine for 96 h. Data represent mean ± SEM (*n* = 4), percentage of 100 mg·dL^−1^ d‐glu. *P < 0.01, vs. 100 mg·dL^−1^ d‐glu. **P < 0.05, vs. 450 mg·dL^−1^ d‐glu. ***P < 0.01, vs. 450 mg·dL^−1^ d‐glu.

We next examined the effects of ARBs and CCBs on the high glucose‐induced *CYP11B2* transcription. As shown in Fig. 8A, each ARB at 1 μmol·L^−1^ did not affect the high glucose effect. In contrast, each CBB dose dependently inhibited the high glucose‐induced *CYP11B2* transactivation (Fig. 8B). It is therefore suggested that calcium channels, but not AII type 1 receptor, are involved in the high glucose effect.

## Discussion

In this study, we first demonstrated the stimulatory effect of high glucose on *CYP11B2* transcription and mRNA expression as well as aldosterone secretion in human adrenal cells (Figs 1 and 3A). The high glucose‐induced *CYP11B2* mRNA expression was not observed when we used glucose analogs, 2‐deoxy‐d‐glu and 3‐*O*‐methyl‐d‐glu 15, 16 (Fig. 7), suggesting that it is necessary for d‐glu to be metabolized within the cells for the stimulatory effect. As we also observed the high glucose‐induced *StAR* mRNA expression (Fig. 2E), increased *StAR* and *CYP11B2* may coordinately induce aldosterone production. Aldosterone not only induces hypertension and vascular damage in combination with sodium 5, but is also known to inhibit glucose‐induced insulin secretion in pancreatic ß‐cells 18 as well as insulin signaling in peripheral tissues 19, 20. Therefore, the high glucose‐induced aldosterone may induce ‘a vicious cycle’ in terms of the exacerbation of glucose intolerance/diabetes mellitus. Although the plasma aldosterone concentration in patients with diabetes has long been controversial, it has recently been confirmed to be significantly higher than that of normal subjects by fixing sodium/potassium intake and the time for drawing blood samples 21. Therefore, based on our present observation, high glucose‐induced aldosterone production may possibly contribute to the increased plasma aldosterone level in patients with diabetes.

Transient transfection experiments using *CYP11B2* 5′‐flanking region deletion mutants and NBRE‐1 point mutant revealed that the NBRE‐1 element, which is known to be activated by NURR1/NGFIB binding 11, 14, was responsible for the high glucose‐induced *CYP11B2* transactivation (Fig. 4). Additionally, high glucose was demonstrated to induce the mRNA expression of NURR1 significantly compared with that of NGFIB (Fig. 5). However, as NURR1 knockdown did not affect the high glucose‐induced *CYP11B2* mRNA expression (Fig. 6), other NR4A family members or other transcription factors may bind to and activate NBRE‐1 element to induce *CYP11B2* transactivation. In human adrenocortical neoplasms, *CYP11B2* mRNA expression significantly and positively correlated with NURR1 mRNA expression, but not with NGFIB mRNA expression 22. As H295R cells are also derived from human adrenocortical carcinoma, it is plausible that NURR1 also plays an indispensable role in *CYP11B2* transactivation in the cells. Interestingly, high glucose was demonstrated to suppress SF‐1 mRNA expression, although not significantly (Fig. 5C). As SF‐1 is known to suppress *CYP11B2* transcription 23, 24, the high glucose‐mediated SF‐1 decrease may also contribute to the induction of *CYP11B2* transactivation. The mechanisms by which high glucose induces NURR1 mRNA expression remain uncertain. AII and potassium are two major factors that regulate *CYP11B2* transcription 14, 25. AII is known to bind to AII type 1 receptor and activate phospholipase C to increase inositol 1,4,5‐trisphosphate (IP_3_), and IP_3_ induces the release of intracellular calcium from the endoplasmic reticulum, while potassium causes depolarization of the membrane allowing extracellular cytoplasmic calcium influx through the T‐ and L‐type calcium channels 14, 25. In both cases, increased calcium leads to the activation of calcium/calmodulin‐dependent kinase, resulting in the induction of NURR1 mRNA expression 14, 25. When we treated the stable *CYP11B2*‐H295R cells with several ARBs, the high glucose‐induced *CYP11B2* transactivation was not affected (Fig. 8A). In contrast, when we treated the cells with several CCBs, they, especially benidipine that blocks both T‐ and L‐type calcium channels 26, dose dependently inhibited the high glucose‐induced *CYP11B2* transactivation (Fig. 8B). These data indicate that high glucose may affect at least pathway(s) mediated via T‐ and/or L‐type calcium channels, but not pathway(s) mediated via AII type 1 receptor. Interestingly, we have recently observed the high glucose‐induced mRNA expression of T‐type calcium channel subunits (CaV3.1, CaV3.2, and CaV3.3) 27, which may also be involved in the high glucose effect. Further studies are needed to clarify the precise molecular mechanisms of the high glucose‐induced *CYP11B2* transactivation.

In summary, we here demonstrated high glucose‐induced *CYP11B2* transcription and mRNA expression as well as aldosterone secretion via NURR1 induction. As our observation provides a novel insight into the etiology of hypertension in patients with diabetes, it may also lead to novel therapeutics, such as an inhibitor of *CYP11B2* transcription, for patients with diabetes complicated with hypertension.

## Author contributions

AU, AY, and AS conceived and designed the experiments. HS, NK, EN, KS, IS, KS, MK, DS, and TSI performed the experiments. MK, RP, TSI, AU, ASH, WER, and AY analyzed the data. WER contributed reagents/materials/analysis tools. HS, NK, TSI, SI, AY, and AS wrote the manuscript.
